# Use of Cumulative Poisson Probability Distribution as an Estimator of the Recombination Rate in an Expanding Population: Example of the *Macaca fascicularis* Major Histocompatibility Complex

**DOI:** 10.1534/g3.111.001248

**Published:** 2012-01-01

**Authors:** Antoine Blancher, Alice Aarnink, Nicolas Savy, Naoyuki Takahata

**Affiliations:** * Laboratoire d'Immunogénétique Moléculaire, EA 3034, Faculté de Médecine Purpan, Université Paul Sabatier, Toulouse 3, CHU de Toulouse, 31059 Toulouse cedex 9, France; † Institut de Mathématiques, Université Paul Sabatier, Toulouse 3, 31059 Toulouse cedex 9, France; ‡ Graduate University for Advanced Studies (Sokendai), Hayama, Japan

**Keywords:** Poisson distribution, recombination rate, expanding population, major histocompatibility complex, MHC, *Macaca fascicularis*

## Abstract

We describe a method to estimate the rate of recombination per generation from the genotypes of a large individual sample of an expanding population, for which the founding event is dated. The approach is illustrated with an application to estimating the major histocompatibility complex (MHC) recombination rate in the Mauritian macaque population. We genotyped 750 macaques by means of 17 microsatellites across the MHC region and reconstructed the seven most frequent haplotypes assumed to represent the founding haplotypes (H_rec(0)_) as well as the 31% recombinant haplotypes (H_rec(h)_) resulting from a variable number “h” of recombinations between the founding haplotypes. The relative frequencies of the various classes of haplotypes (H_rec(0)_ and H_rec(h)_) follow a Poisson distribution. By using a maximum likelihood method, we calculated the mean of the Poisson distribution that best fits the data. By dividing this mean by the number of generations (50−100) from the date of the population founding, we deduced that rate of recombination in the MHC is approximately 0.004 to 0.008 in the Mauritian macaque population. When the founding date of the population is precisely known, our method presents a useful alternative to the coalescent method.

The major histocompatibility complex (MHC) is one of the most polymorphic regions in human as well as in nonhuman primates and other mammal species studied. This high degree of polymorphism is particularly adapted to the study of recombination in a relatively short region of the genome. The human MHC spans approximately 3.3 Mb on the short arm of chromosome 6 and contains the most polymorphic loci (and, consequently, haplotypes) known in humans. Approximately 40% of the 128 genes in this region participate in immune responses (The MHC Sequencing Consortium, 1999). It is strongly suspected that the long-term persistence of certain MHC haplotypes is favored by their positive selection because of beneficial interactions between specific allelic products of the different loci composing these stable haplotypes during a long period (Beck and Trowsdale 2000).

The recombination events across the MHC have been extensively studied in humans, first by the studies of recombination in families and more recently by high-resolution mapping of recombination events in single sperm (Cullen *et al.* 2002) and through high-resolution recombination maps inferred from high-density single nucleotide polymorphism data (Wegmann *et al.* 2011).

In the present study, we sought to estimate the rate of recombination inside the MHC of cynomolgus monkeys (*Macaca fascicularis*) originated from the Mauritius Island. The Mauritian macaque population was founded approximately 400 years ago by the introduction of a few animals via Portuguese or Dutch sailors. The strong impact of the founding bottleneck was clearly confirmed by mitochondrial DNA analysis (Lawler *et al.* 1995) as well as by the study of the polymorphism of several nuclear genes. We have estimated that the most probable number of animal founders was around 12 (Bonhomme *et al.* 2008). These animals were most likely captured in Java and/or Sumatra (Kondo *et al.* 1993, Tosi and Coke 2007). They were released in the wild on the Mauritius Island, where they reproduced so rapidly that 100 years after their introduction, the macaques have pullulated on the island, forcing the farmers to leave because of the environmental and agricultural damage caused by the macaques (Sussman and Tattersall, 1981, 1986). From the historical records, macaques were imported only once to the island. The Mauritian macaque population, then, is an archetype of isolated insular population. It constitutes a model of a population with a strong founding effect, followed by a rapid expansion and a population size plateau of approximately 50,000 animals corresponding to the current estimated population size. There is no record of severe episodes of bottleneck after the founding event.

In a study of a large population sample (N = 750 unrelated animals) genotyped by means of 17 microsatellites across the MHC region, we reconstructed the MHC founding haplotypes in the Mauritian macaque population (Aarnink *et al.* 2011, Wiseman *et al.* 2007). The definition of ancestral haplotypes allowed us to detect and characterize recombinant haplotypes. From the frequencies of recombinant haplotypes, we determined a new method to estimate the recombination rate inside the MHC region and compared this rate with that obtained by the coalescent method.

## Material and Methods

### Animals

The animals belonged to several Mauritius breeding companies (Le Tamarinier, Noveprim). Some animals (20%) were captured in the wild, whereas the others were F1 animals born in captivity from animals captured in the wild. Animals born in captivity have neither their parent in common (as attested by the records of the breeding companies) and were therefore considered unrelated. The animals bleeding were performed following the ethical rules of animal handling.

### Genomic DNA samples

Genomic DNA was extracted from peripheral blood sample using either QIA amp Blood Kit (QIAGEN, Courtaboeuf, France) or a classical phenol–chloroform method.

### MHC genotyping

**Figure 1:**
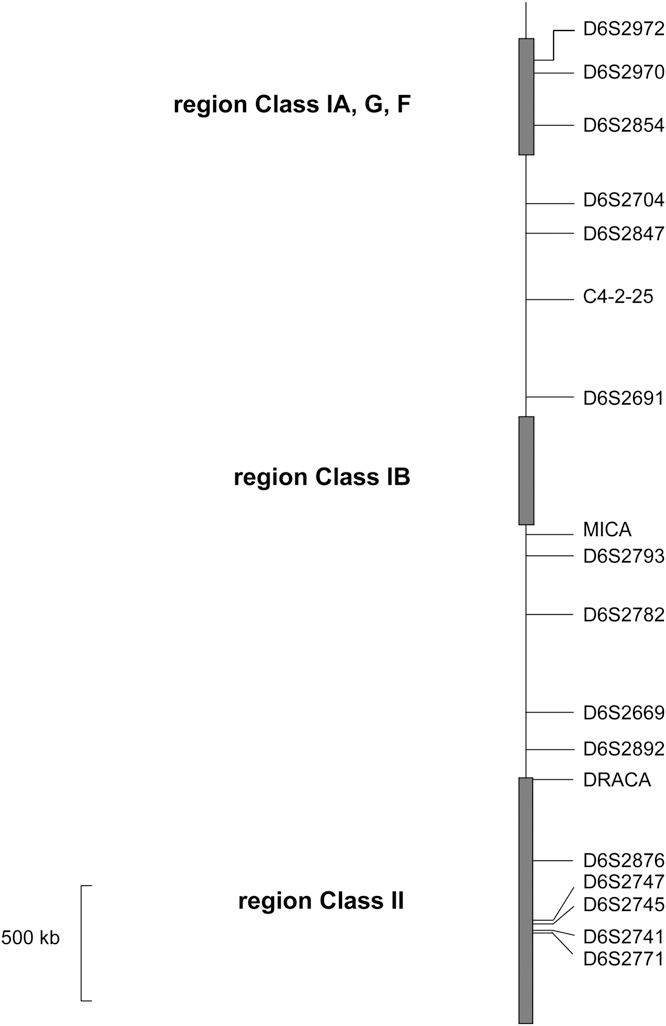
Localization of microsatellite markers on the MHC map of cynomolgus macaque. The regions of class-IA, -IB, and -II genes are shown as boxes (telomere is at the top of the diagram). The markers are positioned by reference to the sequence data (Watanabe *et al.* 2007; and T. Shiina, personal communication).

The MHC genotype was determined with 17 microsatellites scattered across the MHC region; the primers used are described in the supporting information, Table S1 (Aarnink *et al.* 2011, Bonhomme *et al.* 2005, Wiseman *et al.* 2007). Figure 1 describes the locations of the microsatellite markers on the genetic map of the cynomolgus macaque MHC. Genotypes were determined with DNA Size Standard-kit- 600 (Beckman Coulter, Villepinte, France) after denaturation and separation of the amplification products by capillary electrophoresis with a CEQ8000 analyzer and scored with the software CEQ8000 Genetic Analysis System v8.0 (Beckman Coulter, Villepinte, France).

#### Haplotype reconstruction:

First, we deduced the most frequent haplotypes from homozygous animals. The other haplotypes were deduced by the study of heterozygous animals having one of the most frequent haplotypes. In this way, we characterized seven haplotypes, thereafter referred to as the founding haplotypes, similar to those previously described (Mee *et al.* 2009, Wiseman *et al.* 2007). Thereafter, for each animal, we reconstructed the two most probable haplotypes. This individual haplotyping was carefully checked to define the most parsimonious reconstruction (*i.e.* the one that requires the minimum number of recombination of the seven ancestral haplotypes). Because the number of alleles detected in the population for each microsatellite is frequently lower than the number of founding haplotypes, the points of recombination are not always precisely defined (see Table 1 for the interhaplotype allele sharing). The haplotyping of the 750 animals obtained by this method was confirmed by means of Phase software (Stephens *et al.* 2001, Stephens and Donnelly 2003).

**Table 1:** Description of the seven MHC founding haplotypes defined in the Mauritian cynomolgus macaque population by means of 17 microsatellites

Microsatellites	The Seven Founding Haplotypes of the Mauritian Macaque Population						
	H1	H2	H3	H4	H5	H6	H7
*D6S2972*	129	121	123	121	121	123	123
*D6S2970*	303 (307*^a^*)	307 (303*^a^*)	295	315	311	353	311
*D6S2854**^b^*	173−193	173−193	173−193	173−193−197	213	173−193−197	173−193
*D6S2704*	163	163	145	147	153	147	161
*D6S2847*	321	321	321	321	321	323	323
*C4-2-25*	235	237	235	235	235	235	235
*MICA*	203	200	200	200	206	194	203
*D6S2793*	276	268	246	246	246	246	278
*D6S2782*	324	343	337	341	341	337	337
*D6S2669*	130	112	143	130	110	97	110
*D6S2892*	208	202	206	202	205	208	202
*DRACA*	237	268	272	266	266	268	270
*D6S2876*	215	211	217	211	204	211	211
*D6S2747*	208	203	Null or 194*^c^*	191	206	210	191
*D6S2745*	299	311	303	303	299	299	303
*D6S2771*	398	399	399	395	398	399	395
*D6S2741*	266	276	256	258	268	272	258

a Most of the haplotypes H1 and H2 have the alleles 303 and 307, respectively. However, the allele 307 is observed also in 10 of 273 H1 haplotypes and the allele 303 is also present in 19 of 186 H2 haplotypes. Because the microsatellite *D6S2970* is based on a tetranucleotide repeat, we have interpreted the allele variations in haplotypes H1 and H2 as consequences of mutations.

b As for the microsatellite *D6S2854*, fragments of respective lengths 173 and 193 bp were amplified in all animals but homozygous H5/H5. In these animals a single 213-bp fragment was amplified. The 197-bp allele was observed only in animals with haplotype H4 or H6.

c A fragment of 194 bp was amplified weakly from all DNA samples from H3/H3 homozygous animals. However, this fragment was never obtained in the H3 heterozygous animals.

### Statistical method

#### Estimation and confidence interval of the intensity:

The intensity of a Poisson distribution, which equals the mean of the distribution, is denoted by the Greek letter λ and can be estimated from observed data with a maximum likelihood procedure. It is usual to consider that the maximum likelihood estimation of λ, denoted by $\hat{\lambda }$, is the mean of the observed values (Van der Vaart 1998). Moreover, by taking into account the asymptotic normality of the maximum likelihood estimator and applying the Slutsky’s theorem (Van der Vaart 1998), we obtained the following expression of the confidence interval of λ for a given confidence level α:

$$\lambda \in \left[\hat{\lambda }-u_{\alpha /\text{2}}\times \sqrt{\frac{\hat{\lambda }}{n}},\hat{\lambda }+u_{\alpha /\text{2}}\times \sqrt{\frac{\hat{\lambda }}{n}}\right],$$

Where *u*_a/2_ is the fractile of the standard normal distribution and *n* the number of observations.

#### Quality of fitting:

To evaluate the quality of fitting between observed data and the Poisson distribution of intensity λ denoted by *P*(λ), we applied an usual χ^2^ test (Dacunha-Castelle and Duflo 1986).

Consider a sample $\left(x_{1},x_{2},\ldots ,x_{n}\right)$.

The hypotheses are:

$H_{\text{0}}$: $\left(x_{\text{1}},x_{\text{2}},\ldots ,x_{n}\right)$ comes from a P(λ) distribution,

$H_{\text{1}}$: $\left(x_{\text{1}},x_{\text{2}},\ldots ,x_{n}\right)$ does not come from a P(λ) distribution.

Consider $n_{i}^{th}$ the expected value under the hypothesis $H_{0}$given by

$$n_{i}^{th}=n\times \sum_{k=p_{i}}^{p_{i+1}}\frac{e^{-\lambda }\lambda^{k}}{k!},$$

where *p*_0_ = 0 and *p*_i_ is such that for all *i*, $n_{i}^{th}\geq \text{5}$and $n_{i}^{obs}$are the corresponding number of observed values, *i.e.* this means the number of $x_{k}$ such that $p_{i-\text{1}}\leq x_{k}<p_{i}$. By merging the bins in this way, we ensure that $n_{i}^{th}\geq 5$ for every *i*, a necessary condition for the validity of the χ^2^ test.

Denote by *p* the number of such values. The statistic of the test is given by:

$$\chi_{c}^{\text{2}}=\sum_{i=\text{1}}^{p}\frac{{\left(n_{i}^{obs}-n_{i}^{th}\right)}^{2}}{n_{i}^{th}},$$

and the decision is taken by the use of a *P* value calculated from a χ^2^ (*p* − 2) distribution. The degree of freedom *p*-2 is the number of bins − 1 minus 1 because we have estimated the parameter of the Poisson distribution.

### Estimation of recombination rate by the coalescent method

To estimate the recombination rate by the coalescent method, we used the Phase software (Crawford *et al.* 2004, Li and Stephens 2003). We used the recombination model (-MR0 option), the general model for recombination rate variation in Li and Stephens (2003). As recommended, we used the -X option to obtain more accurate estimates.

For each interval between two consecutive markers, we obtained the value of ρ = 4N_e_c. We calculated, for each interval, the rate of recombination per generation by dividing ρ by 4N_e_. As the recombination rates are lower than 3% between two consecutive markers, we summed all values of “c” to deduce the rate of recombination on the complete MHC region analyzed here.

To estimate the N_e_ in the Mauritian macaque population, we applied the classical logistic growth model of Verhulst (equation hereafter) and calculated the harmonic mean of the population size from the founding event to now (50−100 generations depending on the mean generation time, 4−8 years) in the Mauritian macaque population.

$$N_{t}=\frac{N_{0}\cdot K}{N_{0}+\left(K-N_{0}\right)\cdot e^{\left(-r_{0}.t\right)}}$$

Where *N*_t_ number of animals at a given generation;
*N*_0_ number of animal founders;
*K* the maximum number of animals;
*r* the intrinsic growth rate; and
*t* time in generation.

As for *K*, we have estimated that the maximum number of animals of a given generation was one-quarter of the total population size at a given time. Therefore, with a current total population size of approximately 48,000, we obtain a reasonable estimate of 12,000 individuals in a given generation.

It is noteworthy that the Verhuslt model does not take into account the macaque reproduction peculiarities (preference for dominant male and the partitioning among female lineages) that tend to reduce the effective population in comparison with the real population size.

## Results

We studied genotyped 750 animals randomly chosen and born from different breeders. The genotypes were characterized for 18 microsatellites across the MHC region (Figure 1). See File S1 for the allelic data set from these experiments. One microsatellite (*D6S2691*) was found instable with frequent mutations (insertion or deletion of one repeated unit) and was therefore discarded from further analyses (data not shown). The mutations of the microsatellites retained for the haplotype analysis were infrequent (Table 1 and Table 2). By the analysis of these genotypes, we reconstructed seven founding haplotypes (as described in *Material and Methods* and Table 1).

**Table 2:** List of rare microsatellite alleles (F < 1%) observed in the sample of 750 Mauritian macaques

Microsatellite	Allele	Number
*D6S2972*	127	1
*D6S2970*	299	3
	319	8
	357	8
*D6S2854*	181	1
	193	1
*D6S2704*	165	1
*D6S2691*	242	1
	247	9
	253	6
	255	8
	263	1
	271	3
	279	2
	303	1
	305	1
*D6S2793*	280	1
*D6S2892*	212	1
	216	1
*D6S2741*	265	1
	274	2
	278	5

**Figure 2:**
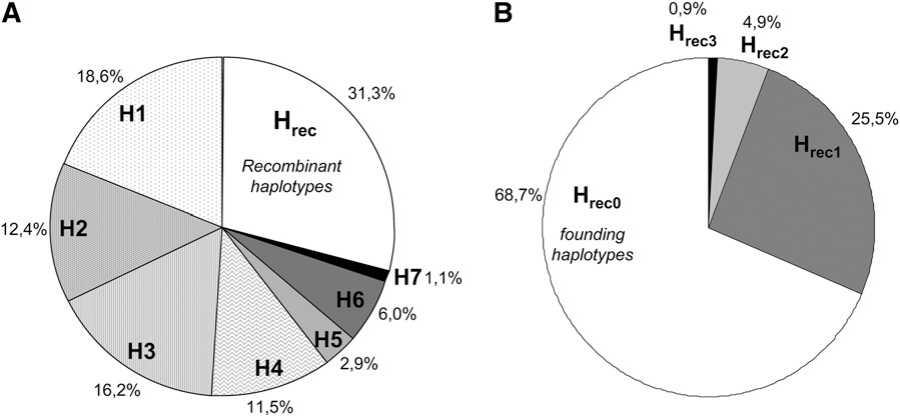
MHC haplotype frequencies in the Mauritian cynomolgus macaque population. (A) The frequencies of the seven most frequent haplotypes (founding haplotypes) and of the recombinant haplotypes in the 750 animals studied here. (B) The frequencies of intact and recombinant MHC haplotypes observed in our sample. The haplotypes are grouped in categories as a function of the number of recombinations they present: intact haplotypes (H_rec0_) have no recombination, and recombinant haplotypes (H_rec(h)_) can be deduced from founding haplotypes by assuming a variable number (h = 1−3) of recombinations

The frequencies of founding haplotypes, called H1, 2, 3, 4, 5, 6, and H7, are similar to those reported previously by others (Budde *et al.* 2010, Mee *et al.* 2009, Wiseman *et al.* 2007) and by us (Aarnink *et al.* 2011) (Figure 2).

As described in *Material and Methods*, for each animal, we retained systematically the most parsimonious haplotype reconstruction (the reconstruction that requires the minimum number of recombination of ancestral haplotypes). The haplotypes can be grouped in various categories as a function of the number of recombinations presented: (1) intact haplotypes (H_rec(0)_) have no recombination (*i.e.* they are identical to founding haplotypes); and (2) recombinant haplotypes (H_rec(h)_) can be deduced from founding haplotypes by assuming a variable number (h) of recombinations. Several examples of recombinant haplotypes are given in the Figure S1.

We found 187 different types of recombinant haplotypes (114, 61, and 12 types of H_rec1_, H_rec2_, and H_rec3_, respectively). The relative frequencies of the most frequent recombinant haplotypes were lower than the frequency of the haplotype M7, the rarest Mauritian ancestral haplotype. We considered therefore that all recombinant haplotypes observed in our animal sample arose after the founding of the Mauritian population (*i.e.* that none of the recombinant haplotypes was brought by the founding animals).

**Figure 3:**
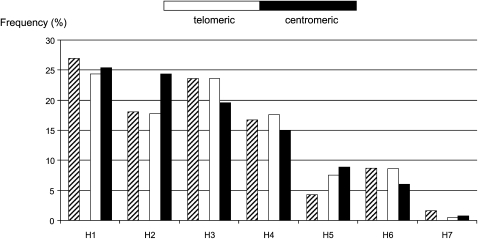
Haplotype frequency distribution observed at the two ends of haplotypes having a single recombination. The frequency distribution of founding haplotypes observed at the telomeric and centromeric ends of the 382 haplotypes having a single recombination is compared with the relative frequency distribution of the ancestral haplotypes in the sample (hatched bars, N = 1030). The frequency distribution at the telomeric end of recombinant haplotypes does not differ from the distribution of founding haplotypes (χ^2^ *P* = 0.56) whereas the frequency distribution at the centromeric end of recombinant haplotypes differed slightly (although significantly, χ^2^ *P* = 0.0003).

**Figure 4:**
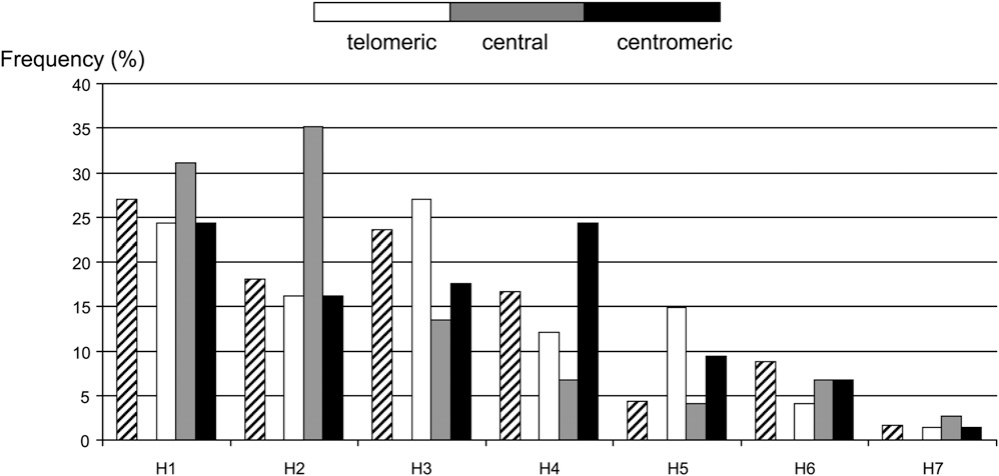
Haplotype frequencies observed in the three parts of haplotypes presenting two recombinations. The white, gray, and black bars represent, respectively, the frequencies of haplotypes in the “telomeric,” “central,” and “centromeric” regions of 74 recombinant haplotypes presenting two recombinations. The hatched bars correspond to the frequencies of the ancestral haplotypes (N = 1030). The frequency distributions of the telomeric and centromeric ends of double recombinant haplotypes are similar to those of intact MHC haplotypes (χ^2^ *P* = 0.56 and *P* = 0.39, respectively) whereas the frequency distribution in the “central” region differ significantly from the distribution of the founding haplotypes (χ^2^ *P* = 0.001).

As for the single recombinant haplotypes, we have studied the relative frequencies of the haplotypes at both sides of the recombination point. The frequency distributions for the telomeric and centromeric parts of the recombinant haplotypes are very close to the frequency distribution of the nonrecombinant haplotypes (Figure 3). As for double recombinant haplotypes, the frequency distributions of the telomeric and centromeric parts do not differ from the distribution of founding haplotypes, contrary to the distribution of haplotype frequencies between the two recombination points (Figure 4).

Assuming that recombinations are rare events, we hypothesized that their accumulation in the Mauritian population was compatible with the following assumptions:

- the probability of recombination is constant from the founding event up to the current time;
- the probability of a recombination is not influenced by a previous recombination events; and
- the probability for a given haplotype to have not been transmitted to the current population does not depend on the number of recombinations experienced by this haplotype (*i.e.* the recombinations are strictly neutral).

Moreover, we assume that recombination identified within a given animal resulted from an event independent of those resulting in identical recombinant haplotypes observed in other animals. This assumption is not unlikely because of the large growth of the macaque population during the past 400 years from a very small founder population, which reduces the amount of shared ancestry concerning the recombinant haplotypes. Under all these assumptions, one can deduce that the numbers (or frequencies) of haplotypes presenting no or a given number of recombinations follow a Poisson distribution.

To check this latter proposal, we compared the observed relative frequencies distribution H_rec(0,1,2,3,h)_ to those deduced from a Poisson distribution with an intensity estimated from the observed data by a maximum likelihood procedure (see *Materials and Methods* for details). The intensity of the Poisson distribution deduced from the observed frequencies H_rec(0,1,2,3,4)_ was 0.381, and with a risk of 5%, we can say that the intensity was comprised between 0.351 and 0.413 (Table 3). Moreover, the statistical comparison by the χ^2^ test allows us to say there is no significant difference between the distribution of the observations and the values calculated from a Poisson *P*(0.381) distribution (the calculated value is $\chi_{c}^{2}=1.436$ and the associated *P*-value *P* = 0.488). Therefore, assuming that the distribution follows the optimized Poisson distribution, the intensity of this distribution represents the frequency of recombination during the period spanning the founding of the Mauritian macaque population to the current time, which can be estimated to 0.381 (± 0.031). If we assume that this period is approximately 400 years and that the mean time generation of *Macaca fascicularis* is between 4 and 8 years, the frequency of recombination per generation is between 0.0038 (± 0.00031) and 0.0076 (± 0.00062).

**Table 3:** Recombinant haplotype numbers observed in the population sample and corresponding numbers expected from the optimized Poisson distribution

Recombination Number Per Haplotype	Numbers of Observations	Haplotype Numbers Expected from a *P* (λ) Distribution with		
		λ = 0.381*^a^*	λ = 0.351*^b^*	λ = 0.413*^c^*
0	1030	1024.77	1055.98	992.49
1	382	390.44	370.65	409.90
2	74	74.38	65.05	84.64
3	14	9.45	7.61	11.65
4	0	0.90	0.67	1.20
5	0	0.07	0.05	0.10

a Intensity of the Poisson distribution estimated by maximum of likelihood from the observed values.

b Lower bound of the confidence interval of the intensity for a risk of 5%.

c Upper bound of the confidence interval of the intensity for a risk of 5%.

In fact, this frequency corresponds to the recombinants detectable by our method. The recombinants events that have occurred in homozygous animals are obviously not detected and are excluded from the deduced frequency. Because of the low number of haplotypes in the population, the frequencies of homozygous animals are not low. In the current population, this frequency is around 10%. We can reasonably estimate that the mean frequency of homozygous animals was relatively stable during the history of the Mauritian macaque population. The frequency of all recombinations in the MHC (detectable or not) can be deduced from the mean frequency of heterozygotes (0.90). The frequency of all recombination events becomes 0.0041 to 0.0082 in function of the mean time generation taken into account (4 and 8 years, respectively).

**Figure 5:**
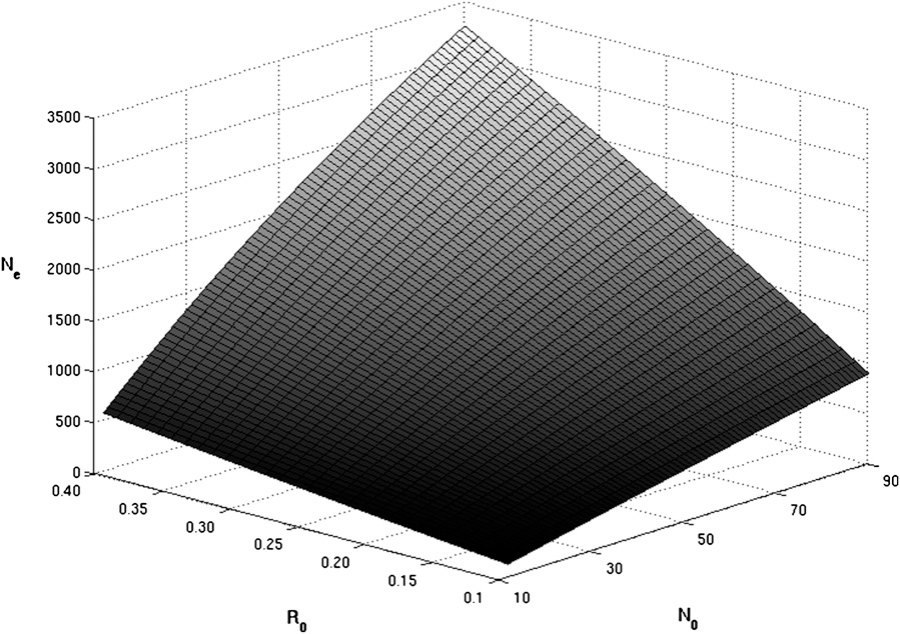
Variation of N_e_ in function of R_0_ and N_0_ under classical logistic growth model of Verhulst. From the time of the macaque population founding up to nowadays (*i.e.* 50−100 generations), the average of N_e_ corresponds to the harmonic mean of the effective population size which was calculated under the assumption of a classical model of logistic growth (see *Materials and Methods* for more details). The figure shows the average N_e_ as a function of R_0_ (0.1−0.4) and N_0_ (10−90) with a constant maximum N_e_ population size (K = 12,000). With a mean generation time of 4 years, the average N_e_ varies from 125 to 3252, as shown in the figure. Under the hypothesis of a generation time of 8 years, the Ne varies from 63 to 1880 (figure not shown).

We decided to estimate the recombination rate by one of the classic coalescent methods. We chose to use the Phase software, which provided the recombination parameter rho = 4N_e_c. To determine the rate of recombination “c” by means of the coalescent method, N_e_ is required. We calculated the N_e_ as the harmonic mean of the effective population size from the time of population founding up to nowadays (*i.e.* 50−100 generations for a mean generation time of 4 or 8 years) under the assumption of a classical model of logistic growth (Verhulst model). Following these calculations, the range of variation of N_e_ estimate is very large (from 63 to 3252) and varies as a function of the demographical parameters: the mean generation time (4 or 8 years), the R_0_ (0.1 to 0.4) and N_0_ (10 to 90; Figure 5). Considering the two extreme estimates of Ne (63 and 3252) and the recombination parameter obtained by means of the Phase software, the rate of recombination in the complete MHC region varies from 0.0027 (N_e_ = 3252) to 0.1365 (N_e_ = 63). The N_e_ that give the recombination rates identical to the estimates obtained by means of the optimized Poisson distribution (0.0041−0.0082) are 1743 or 871, respectively.

## Discussion

By using the distribution of the frequencies of founding and recombinant haplotypes, we estimated the recombination rate per generation in the macaque MHC to be between 0.41% and 0.82%. The rate of recombination deduced by the Poisson distribution of recombinant haplotypes is not incompatible with the very low frequency of recombination observed in pedigree of Mauritian animals bred in captivity. Among 210 informative meioses, only one recombination (0.48%) was observed (E. Mee, personal communication).

The distribution of haplotype frequencies at both sides of recombination point in single recombinant haplotypes is very close to the intact haplotype frequency distribution. This finding suggests that the recombinations were stochastically accumulated along generations. In the central portion of double recombinants, the frequency distribution differs significantly of that of founding haplotypes. This result suggests that the frequency of haplotypes could have varied in the past.

The recombination rate in the macaque MHC seems to be significantly lower that the recombination frequency in the human MHC (human leukocyte antigen), estimated at approximately 3% between HLA-A and HLA-DQ (Cullen *et al.* 2002). Despite this, the macaque MHC differs significantly from that of human by the presence of numerous MHC class I genes (Daza-Vamenta *et al.* 2004, Shiina *et al.* 2006, Watanabe *et al.* 2007) resulting from multiple duplications (Kulski *et al.* 2004), the rate of recombination in macaque MHC is significantly lower than in humans.

In recent studies researchers have revealed that in humans, the rate of recombination in the MHC varies greatly from one individual to another (from 0.8% to 4%) depending on its genotype (Cullen *et al.* 2002). Therefore, it is not impossible that in macaque populations other than that of Mauritius, the mean rate of recombination could differ because of the presence of haplotypes favoring greater rates of recombination. Moreover, our study did not reveal a hot spot of recombination contrary to what was reported in humans. Again this is not incompatible with the low rate of recombination observed in the Mauritian population.

The estimate of the recombination rate deduced from the intensity of the Poisson distribution has a relatively narrow confidence interval. The precision of this estimate depends on the quality of the measure of recombinant haplotype frequencies, and on the incertitude of the mean generation time and of the time elapsed since population founding. As for the mean time of generation, we have taken a relatively large interval (between 4 and 8 years). As for the macaque population founding, most specialists, from historical records, consider that macaques were imported a few decades after the discovery of the Mauritius Island by the Portuguese between 1507 and 1513.

Because the Portuguese were already established in Goa, they took no interest in the isolated Mauritius and did not create a permanent colony there. The island was later rediscovered by Dutch navigators. One of them, Cornelius Matelieff de Jongh, in 1606 was the first to record the presence of monkeys on Mauritius. He wrote: “the only four-footed animals are monkeys” (in Sussman and Tattersall, 1986). At the time of this observation, one can assume that the macaques on the island were abundant enough to retain de Jongh’s attention. The establishment on Mauritius of the monkey population, then, very likely preceded the founding, in 1598, of the tiny Dutch settlement. It is highly probable that the monkeys were introduced by the Dutch or Portuguese sailors at the end of 16th century. Therefore, the time elapsed from the macaque population founding is approximately 400 years. Although various scenarios of macaque population size evolution could be evoked (Bonhomme *et al.* 2008), the simplest one consists in a logistic growth of the population after the founding event characterized by a rapid increase of the population size followed by a plateau of the population size corresponding to the limits of available food on the island. In this model, we must consider that the recombinant haplotypes have accumulated randomly following a rare event law during the time elapsed after the macaques were introduced to the island.

Under these assumptions, we estimated the effective size of the Mauritian population and used it to deduce the rate of recombination from the recombination parameter rho obtained by means of the coalescent method (Phase software). This study reveals a limitation of the coalescent method to estimate the rate of recombination in a population having experienced a rapid growth after a severe founding bottleneck. Indeed, under a logistic Verhulst model, the N_e_ estimate (the harmonic mean of the population size) varies widely (from 63 to 3252) a great incertitude for the recombination rate estimate (from 0.0027 to 0.1365). Note that the interval of recombination rate given by the “Poisson method” is included in the range of the estimate deduced from the coalescent method.

In the comparison between the recombination rate obtained by our method and that obtained by means of Phase, it is important to note that the rho estimate obtained by means of Phase (Li and Stephens 2003) includes “hidden” recombination events that occur when, going backward in time, two lineages recombine and then immediately coalesce with each other. These “hidden” events are by definition not taken into account in our method.

However, the estimate of the recombination rate deduced from the intensity of the Poisson distribution does not take into account the identity by descent of recombinant haplotypes observed in more than one animal in the population sample.

Finally, the new “Poisson method” described here gives a more accurate estimate of the rate of recombination per generation than the coalescent method which requires to estimate also the N_e_.

This method can be applied in populations that have experienced a strong expansion after a severe bottleneck, provided that the time of the founding event and the average time of generation are known. This method is an alternative to the coalescent method when the founding date of the population is precisely known.

## Supplementary Material

Supporting Information
