# Supplementary material for: Use of Cumulative Poisson Probability Distribution as an Estimator of the Recombination Rate in an Expanding Population: Example of the Macaca fascicularis Major Histocompatibility Complex
Source: G3 (Bethesda). 2012 Jan 1;2(1):123–30. doi: 10.1534/g3.111.001248 (PMC3276188; doi:10.1534/g3.111.001248)
Supplement: Supporting Information [file supp_2_1_123__index.html]

Supporting Information 

# Use of Cumulative Poisson Probability Distribution as an Estimator of the Recombination Rate in an Expanding Population: Example of the *Macaca fascicularis* Major Histocompatibility Complex

## Supporting Information for Blancher *et al*, 2012

**Files in this Data Supplement:**

- Supporting Information - Figure S1, Table S1, and File S1
- Figure S1 - Examples of reconstruction of the history of double and triple recombinant haploytpes (PDF, 148 KB)
- Table S1 - Primers used to amplify the 18 MHC microsaellites (PDF, 56 KB)
- File S1 - 750 MHC microsatellite genotypes of Mauritian macaques (.xls, 256 KB)
