# Supplementary material for: Use of Cumulative Poisson Probability Distribution as an Estimator of the Recombination Rate in an Expanding Population: Example of the Macaca fascicularis Major Histocompatibility Complex
Source: G3 (Bethesda). 2012 Jan 1;2(1):123–30. doi: 10.1534/g3.111.001248 (PMC3276188; doi:10.1534/g3.111.001248)
Supplement: Supporting Information [file supp_2.1.123_001248SI.pdf]

Use of cumulative Poisson probability distribution as an estimator of the recombination rate in an expanding population: example of the *Macaca fascicularis* MHC

Antoine Blancher, Alice Aarnink, Nicolas Savy, Naoyuki Takahata

SUPPORTING INFORMATION

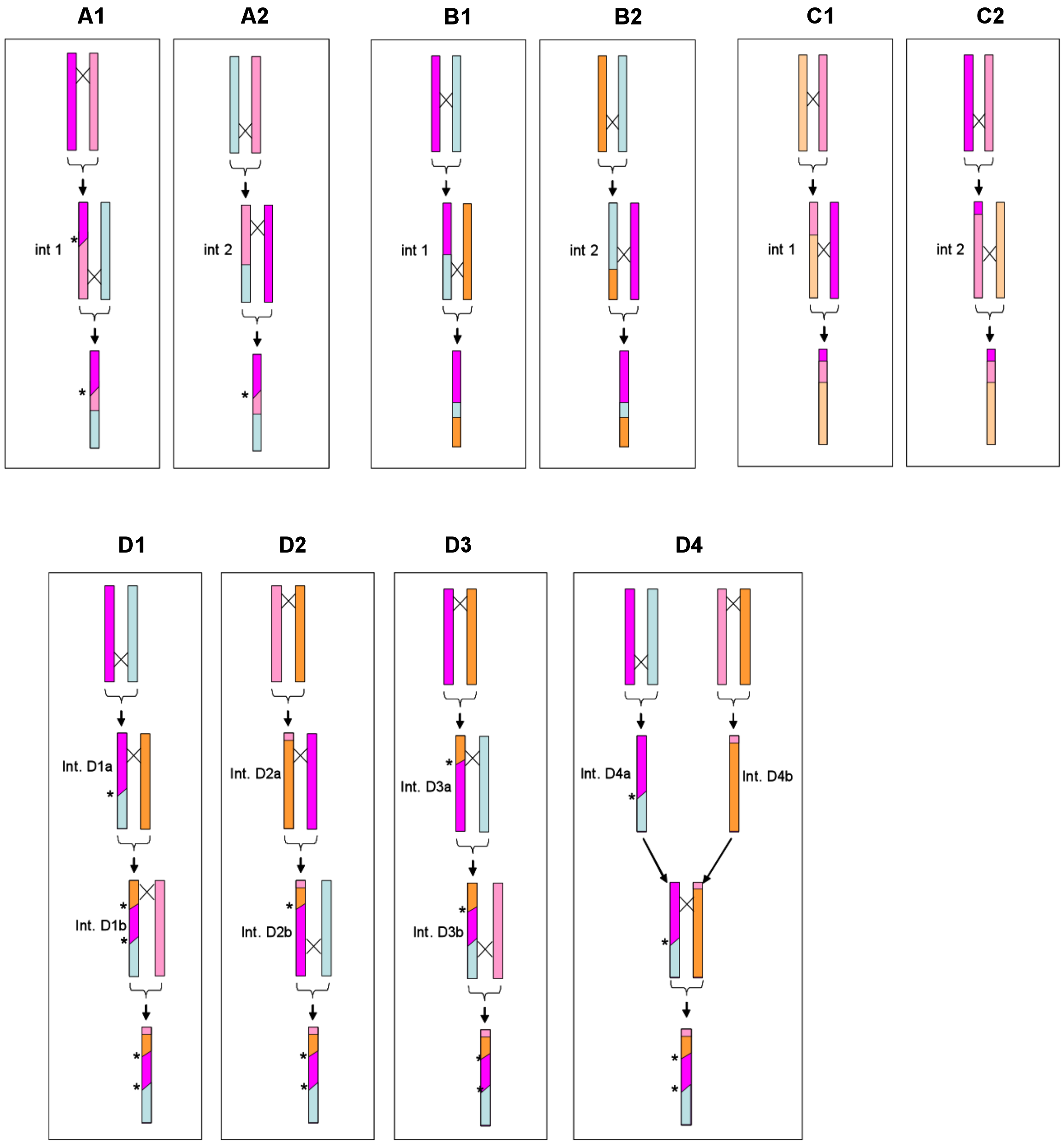

Figure S1 Examples of reconstruction of the history of double and triple recombinant haplotypes. The scenarios of the two recombinations leading to three complex recombinant haplotypes are depicted here (A, B, C). For each recombinant haplotype, the two possible scenarios are presented. It is important to note that all intermediate (int1 or int2) recombinant haplotypes are presents in the 1500 haplotype sample.

When the recombination cannot be localized precisely (haplotype allele sharing) we used a diagonal to delineate the zone of the recombination.

One example of a recombinant haplotype resulting from three recombinations is also given (hypotheses D1 to D4). Three scenarios (D1 to D3) are based on three successive recombinations. In the hypothesis D4 we suppose the recombination of two recombinant haplotypes. Interestingly, all recombinant intermediates (Int. D1b, Int. D2b, Int. D3b) presenting two recombinations (hypotheses D1 to D3) are absents from the population sample. By contrast, the two intermediate recombinant haplotypes (Int. D4a, Int. D4b), of the hypothesis D4 are observed in our sample, rendering the latter scenario the most probable. Obviously, in all hypotheses D1 to D4, three independent recombination events are needed to explain the existence of triple recombinant.

Table S1 Primers used to amplify the 18 MHC microsatellites

Microsatellite	Forward	Reverse	Ref[(a)]
D6S2972	AAATGTGAGAATAAAGGAGA	GATAAAGGGGAACTACTACA	1
D6S2970	TCCCATGGTCAAGTTCTCAG	TCATGGATCTTATCAGCCTC	1
D6S2854	TCATGAGCGTGGCACTGCAC	CCGTATGTTGCAACCAGGAG	1
D6S2704	TTTTGCCACTCTGGAGGATGGG	GAGCATAATATCTGGTCTACTGC	1
D6S2847	TATTGGACAGCACTGCTCTGG	TGCCATTCAGATTGGTTTTTCTG	1
C4-2-25	ATGTTAGTTTTAGAAGATAACACTC	TCTTCTGTGCAAGCAAGCACTGTAC	1
D6S2691	GTAGCTGTGGAAACAGTGTCCATG	CTTGACTTGAAACTCAGAGACC	1
MICA	CCTTTTTTTCAGGGAAAGTGC	CCTTACCATCTCCAGAAACTGC	1
D6S2793	CTACCTCCTTGCCAAACTTGCTATTTGT	AATAGCCATGAGAAGCTATGTGGGGGA	1
D6S2782	TTTACTTGCTCTCACTCTCAGGCC	GGAAGACATTAACTTGTTAGCA	1
D6S2669	TGCCTTCCGTAAGCCTCAGTCT	TTAAGGACAGCAAAGCCAGCAGCA	1
D6S2892	TGCATGTCCTGTGAGGTAAG	ACTCAACCCTGCTGTTGTAG	1
DRACA	TGGAATCTCATCAAGGTCAG	ACATTTGTATGCTTCAGATG	2
D6S2876	GGTAAAATTCCTGACTGGCC	GACAGCTCTTCTTAACCTGC	1
D6S2747	AGGAATCTAGTGCTCTCTCC	CTCTAGCAAAAGGAAGAGCC	1
D6S2745	CCTAGAGATTCCTCCACATTA	CCAATGTTTGATAGCAGACTGGGGT	1
D6S2771	ATTCCTTTCACTAGTTCTGG	CCACTTTAAGAAATTAGAAAAG	1
D6S2741	AGACTAGATGTAGGGCTAGC	CTGCACTTGGCTATCTCAAC	1

[(a)] Reference quoted in the table: (1) WISEMAN *et al.* 2007, (2) BONHOMME *et al.* 2005

File S1

750 MHC microsatellite genotypes of Mauritian macaques

File S1 is available for download as an Excel file at http://www.g3journal.org/lookup/suppl/doi:10.1534/g3.111.001248/-/DC1**.**
