# Supplementary material for: Use of Cumulative Poisson Probability Distribution as an Estimator of the Recombination Rate in an Expanding Population: Example of the Macaca fascicularis Major Histocompatibility Complex
Source: G3 (Bethesda). 2012 Jan 1;2(1):123–30. doi: 10.1534/g3.111.001248 (PMC3276188; doi:10.1534/g3.111.001248)
Supplement: Supporting Information [file supp_2.1.123_FigureS1.pdf]

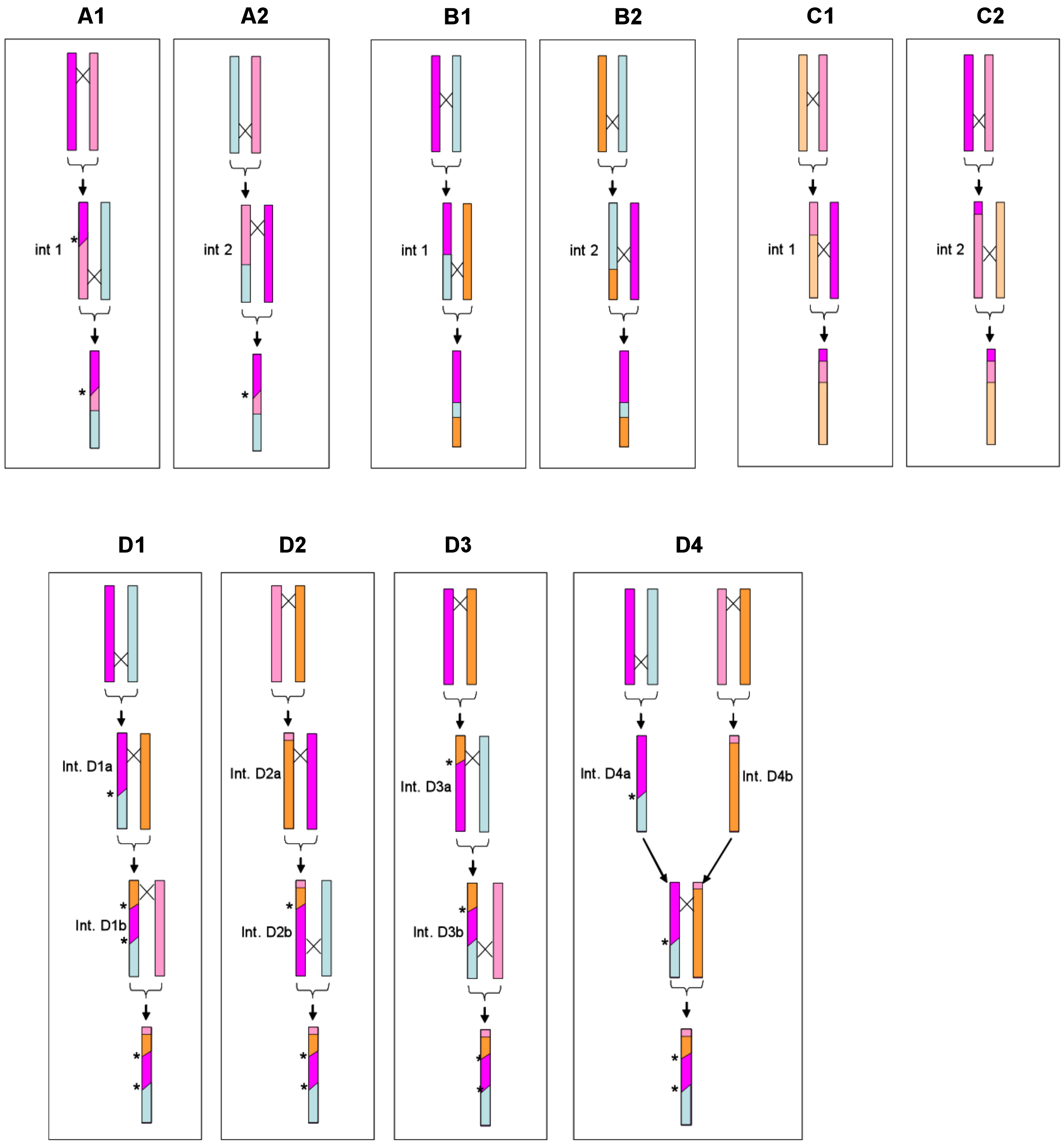

Figure S1 Examples of reconstruction of the history of double and triple recombinant haplotypes. The scenarios of the two recombinations leading to three complex recombinant haplotypes are depicted here (A, B, C). For each recombinant haplotype, the two possible scenarios are presented. It is important to note that all intermediate (int1 or int2) recombinant haplotypes are presents in the 1500 haplotype sample.

When the recombination cannot be localized precisely (haplotype allele sharing) we used a diagonal to delineate the zone of the recombination.

One example of a recombinant haplotype resulting from three recombinations is also given (hypotheses D1 to D4). Three scenarios (D1 to D3) are based on three successive recombinations. In the hypothesis D4 we suppose the recombination of two recombinant haplotypes. Interestingly, all recombinant intermediates (Int. D1b, Int. D2b, Int. D3b) presenting two recombinations (hypotheses D1 to D3) are absents from the population sample. By contrast, the two intermediate recombinant haplotypes (Int. D4a, Int. D4b), of the hypothesis D4 are observed in our sample, rendering the latter scenario the most probable. Obviously, in all hypotheses D1 to D4, three independent recombination events are needed to explain the existence of triple recombinant.
