# Supplementary material for: Use of Cumulative Poisson Probability Distribution as an Estimator of the Recombination Rate in an Expanding Population: Example of the Macaca fascicularis Major Histocompatibility Complex
Source: G3 (Bethesda). 2012 Jan 1;2(1):123–30. doi: 10.1534/g3.111.001248 (PMC3276188; doi:10.1534/g3.111.001248)
Supplement: Supporting Information [file supp_2.1.123_TableS1.pdf]

Table S1 Primers used to amplify the 18 MHC microsatellites

Microsatellite	Forward	Reverse	Ref[(a)]
D6S2972	AAATGTGAGAATAAAGGAGA	GATAAAGGGGAACTACTACA	1
D6S2970	TCCCATGGTCAAGTTCTCAG	TCATGGATCTTATCAGCCTC	1
D6S2854	TCATGAGCGTGGCACTGCAC	CCGTATGTTGCAACCAGGAG	1
D6S2704	TTTTGCCACTCTGGAGGATGGG	GAGCATAATATCTGGTCTACTGC	1
D6S2847	TATTGGACAGCACTGCTCTGG	TGCCATTCAGATTGGTTTTTCTG	1
C4-2-25	ATGTTAGTTTTAGAAGATAACACTC	TCTTCTGTGCAAGCAAGCACTGTAC	1
D6S2691	GTAGCTGTGGAAACAGTGTCCATG	CTTGACTTGAAACTCAGAGACC	1
MICA	CCTTTTTTTCAGGGAAAGTGC	CCTTACCATCTCCAGAAACTGC	1
D6S2793	CTACCTCCTTGCCAAACTTGCTATTTGT	AATAGCCATGAGAAGCTATGTGGGGGA	1
D6S2782	TTTACTTGCTCTCACTCTCAGGCC	GGAAGACATTAACTTGTTAGCA	1
D6S2669	TGCCTTCCGTAAGCCTCAGTCT	TTAAGGACAGCAAAGCCAGCAGCA	1
D6S2892	TGCATGTCCTGTGAGGTAAG	ACTCAACCCTGCTGTTGTAG	1
DRACA	TGGAATCTCATCAAGGTCAG	ACATTTGTATGCTTCAGATG	2
D6S2876	GGTAAAATTCCTGACTGGCC	GACAGCTCTTCTTAACCTGC	1
D6S2747	AGGAATCTAGTGCTCTCTCC	CTCTAGCAAAAGGAAGAGCC	1
D6S2745	CCTAGAGATTCCTCCACATTA	CCAATGTTTGATAGCAGACTGGGGT	1
D6S2771	ATTCCTTTCACTAGTTCTGG	CCACTTTAAGAAATTAGAAAAG	1
D6S2741	AGACTAGATGTAGGGCTAGC	CTGCACTTGGCTATCTCAAC	1

[(a)] Reference quoted in the table: (1) Wiseman *et al.* 2007, (2) Bonhomme *et al.* 2005
